# Ag(I) and Au(III) Mercaptobenzothiazole complexes induced apoptotic cell death

**DOI:** 10.1038/s41598-018-36801-6

**Published:** 2019-01-24

**Authors:** Jositta Sherine, Arun Upadhyay, Amit Mishra, Deepak Kumar, Samanwita Pal, S. Harinipriya

**Affiliations:** 1 Department of Physics and Nanotechnology, SRM Institute of Science and Technology, Kattankulathur, 603203 India; 2 Cellular and Molecular Neurobiology Unit, Department of Biology, Indian Institute of Technology Jodhpur, Jodhpur, Rajasthan 342011 India; 3 Department of Chemistry, Indian Institute of Technology, Jodhpur, Rajasthan 342011 India; 4 Electrochemical Systems Lab, SRM Research Institute, SRM Institute of Science and Technology, Kattankulathur, 603203 India

## Abstract

2-Mercaptobenzothiazole (MBT) complexes of Ag(I) and Au(III) were synthesized by wet chemical method. The structural, optical, ^1^HNMR, ICP – MS and electrochemical studies of the complexes were carried out. The TUNEL assay studies of Ag(I)MBT and Au(III)MBT complexes on A549 cell line indicated induced apoptosis in the cells. TUNEL assay showed 60% cell viability for Ag(I)MBT whereas 80% for Au(III)MBT. Thus Ag(I)MBT can induce cell apoptosis in cells at a higher rate than Au(III)MBT. Therefore these complexes studied here can be a viable option as anti – proliferating agent.

## Introduction

Organometallic compounds such as Ag(I)MBT and Au(III)MBT had been extensively used as medicine due to their anti inflammatory and antibacterial properties. Au salts were used for the treatment of rheumatoid arthritis and Ag for the antimicrobial activity. Preliminary data suggest that the use of Au and Ag complexes for anti-tumour activity which involves direct interaction with DNA as the basis for their cytotoxic effects on tumour cells. Preliminary mechanistic data involved studies on the interaction of selected compounds with plasmid (pBR322) DNA as a model nucleic acid and selected protein kinases (from a panel of 35 protein kinases) with oncological interest^1^. Since the effect of metal complexes on the tumour cells relied on the kinetic behavior and redox stabilities, it is necessary to choose an appropriate ligand donor set (e.g. S and N) to develop a highly stable and effective chemotherapeutic agent^2^. Recently, various thioamides^3^ like 2-mercaptobenzothiazole (MBT), 5-ethoxy-2-mercaptobenzimidazole (EtMBT), 2-mercapto-nicotinicacid(mnaH_2_), 2-mercapto-thiazolidine(mtzdH) and 5-chloro-2-mercapto-benzothiazole (ClMBT) have been used for the synthesis of Au(III) and Au(I) complexes using tetrachloroauric (III) acid (HAuCl_4_) and [Au (tpp)Cl] (tpp = triphenylphosphine(Ph_3_P)). The studies on anti-tumour activity of these complexes against leiomyosarcoma cells showed that ionic complex of Au(III)MBT and Au(I)2-mercapto-thiazolidine exhibited greater anti-tumour activity when compared to cisplatin. In addition, highly sensitive sensing strategy for tetrabromo bisphenol A had been studied recently^4^ via synergetic enhancement of gold nanoparticles and MBT. Apart from anti-tumour studies in cell, MBT nanocomposites were used as drug delivery systems. Controlled release behaviour of halloysite/MBT nanocomposite with calcined halloysite as nanocontainer was studied as drug delivery system in the literature^5^. Solid state differentiation of plasma thiols employing centrifugally activated mercaptobenzothiazole disulphide as an exchange indicator involves the solid state concentration of mono and macromolecular thiols inside the plasma as semi-quantitative indicator to assess various injuries and diseases^6–12^. Recognition and detection of ssDNA was done employing gold electrode modified by MBT self assembled monolayers^13^. This helps to distinguish between ssDNA and dsDNA in biological systems and to reflect the extent of DNA hybridization^13^. Although few studies in the literature^5^ demonstrate Au(III)MBT for anti-tumour activity, Ag(I)MBT complexes are so far not investigated in this context. Thus the present work focuses on the (i) synthesis of Ag(I)MBT and Au(III)MBT complexes via wet chemical method, (ii) structural, optical, electrochemical and ^1^HNMR characterization of the synthesized complexes, (iii) cell viability studies of these complexes on A549 cell lines and (iv) plausible mechanistic pathway for the apoptosis in cells by the complexes.

## Materials and Method

### Synthesis of Tetra Chloro Auric(III) acid (HAuCl_4_)

**Figure 1:**
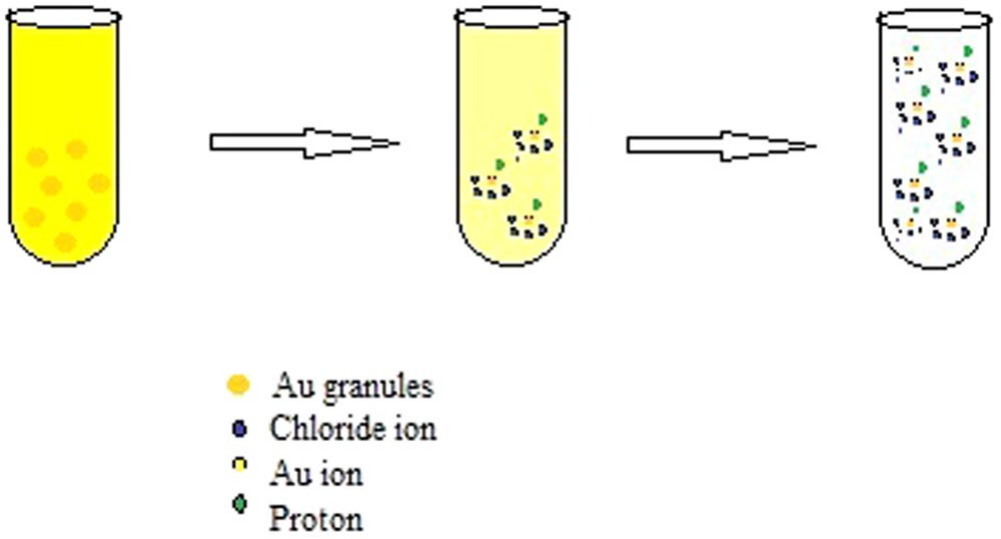
Synthesis of tetrachloroauric acid.

The tetrachloroauric acid (HAuCl_4_) solution (0.01 M) was synthesized by dissolving 2.5 gm of Au granules in 100 ml of aqua regia by continuous stirring and allowed to evaporate until dry. The residue obtained was washed with Conc. HCl and evaporated until dry. This process was repeated 3–4 times. Distilled water was added to the residue and evaporated until dry. This process was repeated twice to obtain crystals^5^ of HAuCl_4_ (Fig. 1).

### Synthesis of Au(III)MBT complex

**Figure 2:**
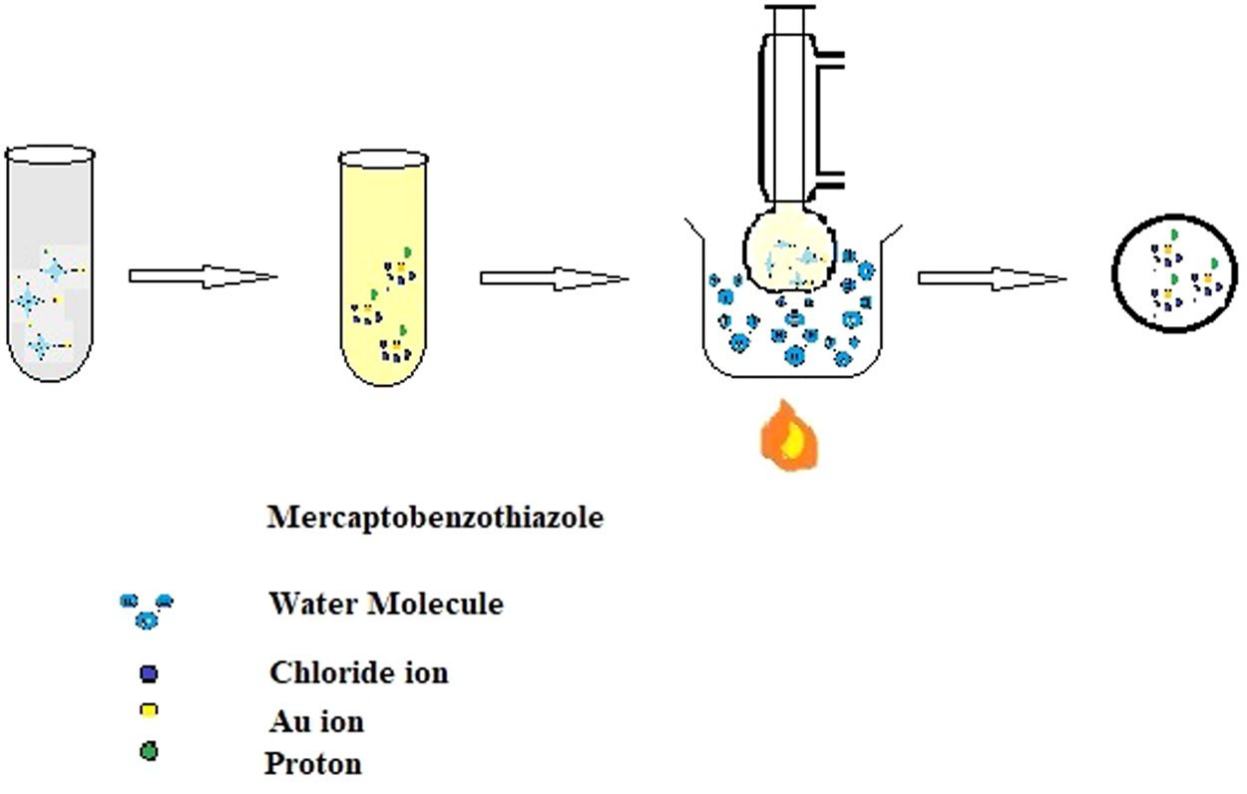
Synthesis of Au(III)MBT crystals from tetrachloroauric acid and MBT.

**Figure 3:**
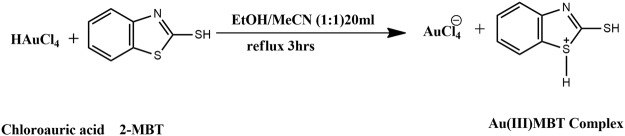
Synthesis of Au(III)MBT complex.

1 mmol commercial MBT (0.167 gm) was dissolved in 10 ml of ethanol. 5 ml of HAuCl_4_ (0.01M) was added to 5 ml of acetonitrile. This solution was then added to the ethanolic MBT and refluxed for 3 hours at 80 °C. After cooling the solution was filtered and the filtrate was kept aside for slow evaporation^3^ to obtain crystals of Au(III)MBT (Figs 2 and 3).

### Synthesis of Ag(I)MBT complex

**Figure 4:**
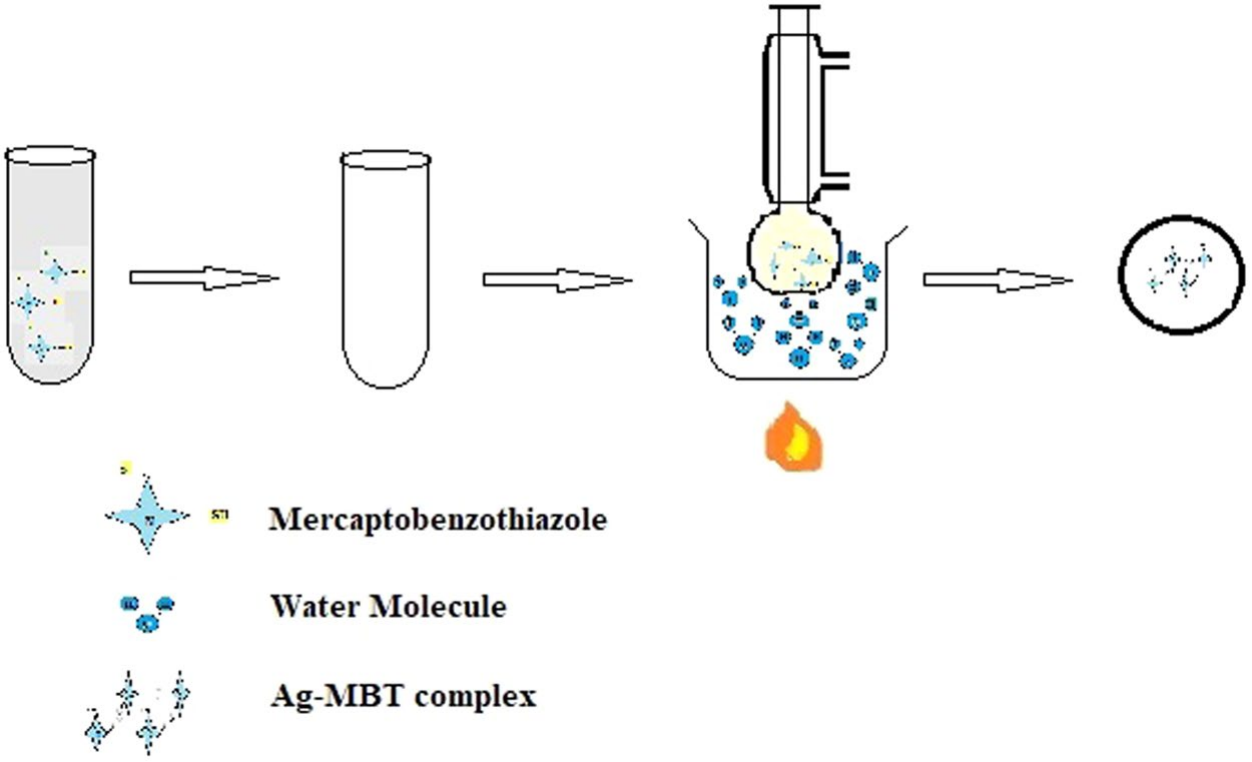
Synthesis of Ag(I)MBT from AgNO_3_ and MBT.

**Figure 5:**
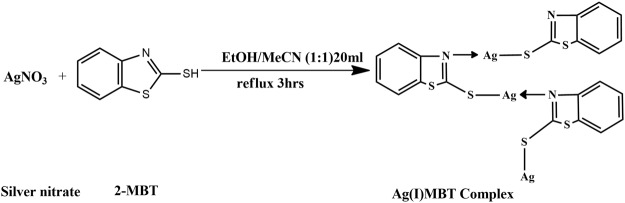
Synthesis of Ag(I)MBT Complex.

1 mmol MBT (0.167 gm) was dissolved in 10 ml of ethanol. 10 ml of acetonitrile containing 0.0835 gm of AgNO_3_ was added to the ethanolic MBT solution and the resulting solution was refluxed for 3 hours at 80 °C. After cooling the solution was filtered and the filtrate was kept aside for slow evaporation to obtain crystals of Ag(I)MBT (Figs 4 and 5).

### Materials Characterization

All solvents used were of reagent grade. MBT and Silver Nitrate(AgNO_3_) were used with no further purification. XRD spectrum was recorded for Ag(I)MBT and Au(III)MBT complexes using BRUKER D8 Advance X-ray diffractometer with Cu Kα source (λ = 1.5406 A°). The UV-Vis absorbance spectrum was obtained by measuring the absorbance using Specord/210, analyticjena UV–Vis spectrophotometer. FTIR spectra were recorded using SHIMADZU, IRAffinity1 spectrometer. Solution state ^1^HNMR spectra of Ag(I)MBT and Au(III)MBT complexes was recorded by Bruker 500 MHz standard bore (SB) NMR spectrometer equipped with BBO probe head. Electrochemical studies of Ag(I)MBT and Au(III)MBT were carried out employing Zahner Zennium electrochemical workstation.

### TUNEL Assay procedure

All cell culture reagents and biochemicals were purchased from Sigma. TUNEL Assay^14^ Kit was procured from Promega. A549 cells were grown in DMEM (Life Technologies, Gaithersburg, Maryland,USA) having 100 μg/ml streptomycin and 10% fetal bovine serum. Cells were platted into six well tissue culture plates for various treatment experiments and on subsequent day at 70–80% confluence.

## Results and Discussion

### Structural Analysis

**Figure 6:**
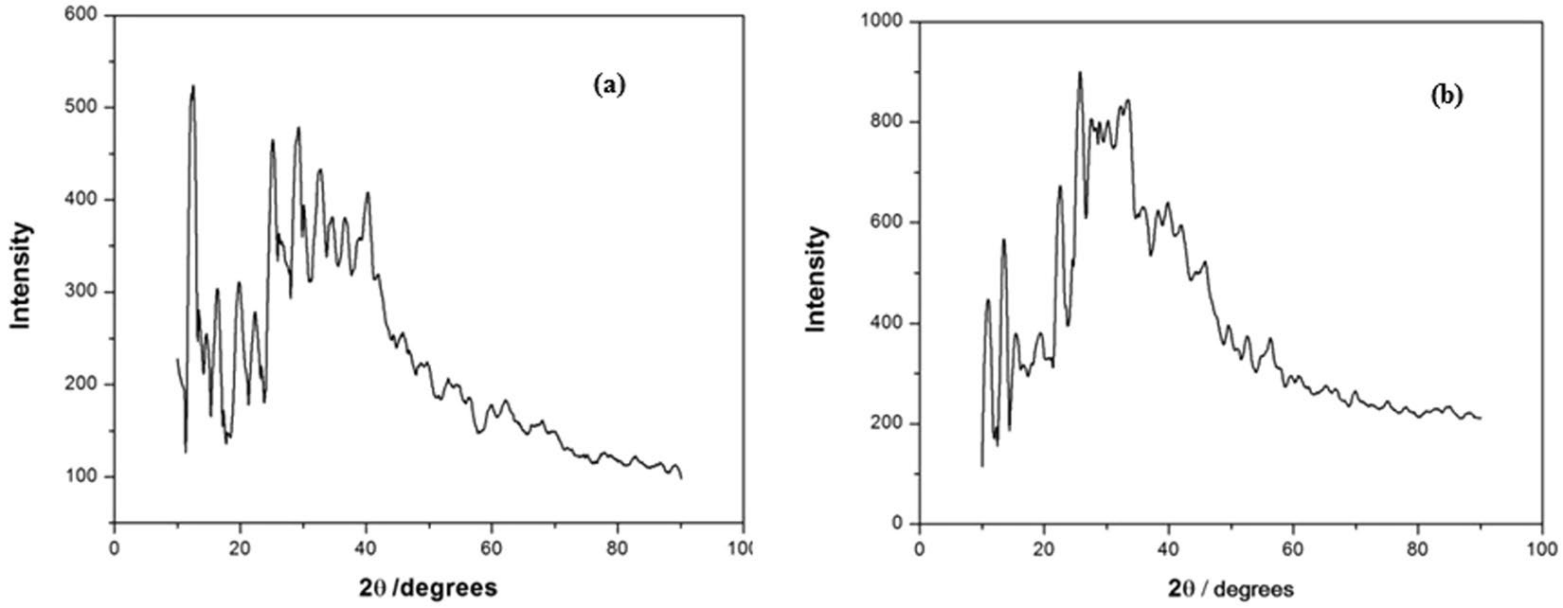
XRD spectra of (**a**) Ag(I)MBT and (**b**) Au(III)MBT complex.

**Figure 7:**
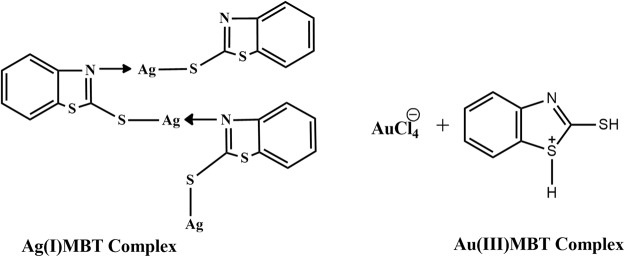
Structure of Ag(I)MBT and Au(III)MBT complexes.

Figure 6 represents the XRD spectra of Ag(I)MBT and Au(III)MBT complexes. The observed XRD spectra is in satisfactory agreement with the literature^5,15–17^. In Fig. 6a,b, the peaks appearing at 11°, 13°, 23°, 24°, 26°, 27° and 28° correspond to MBT (JCPDS No. for MBT: 00-008-0769). For Ag(I)MBT complex, as seen from Fig. 6a, the characteristic peaks for Ag(I) [JCPDS No. for Silver Nitrate: 01-074-1045] appear at 41°, 44° and 55°. The peak broadening from 25° to 40° arises due to the formation of coordination bonding of Ag with hetero nitrogen of MBT as shown in Fig. 7. In Fig. 6b, the characteristic peaks for Au(III) is seen at 38°, 44°. The broadening of the XRD around 20° to 50° is caused due to the presence of ion pair^3^ (Calculated lattice constants for Au(III)MBT (a = 7.9939A°; b = 5.9315A°; c = 15.0911A°) agree satisfactorily with the literature for monoclinic lattice. The d value is calculated as 5 nm and the space group is ‘p21/n’ ref.^3^).

### Optical Analysis

The optical studies of Ag(I)MBT and Au(III)MBT were carried out using UV-Visible and FTIR spectroscopy to understand their optical properties.

#### Electronic spectroscopy analysis

**Figure 8:**
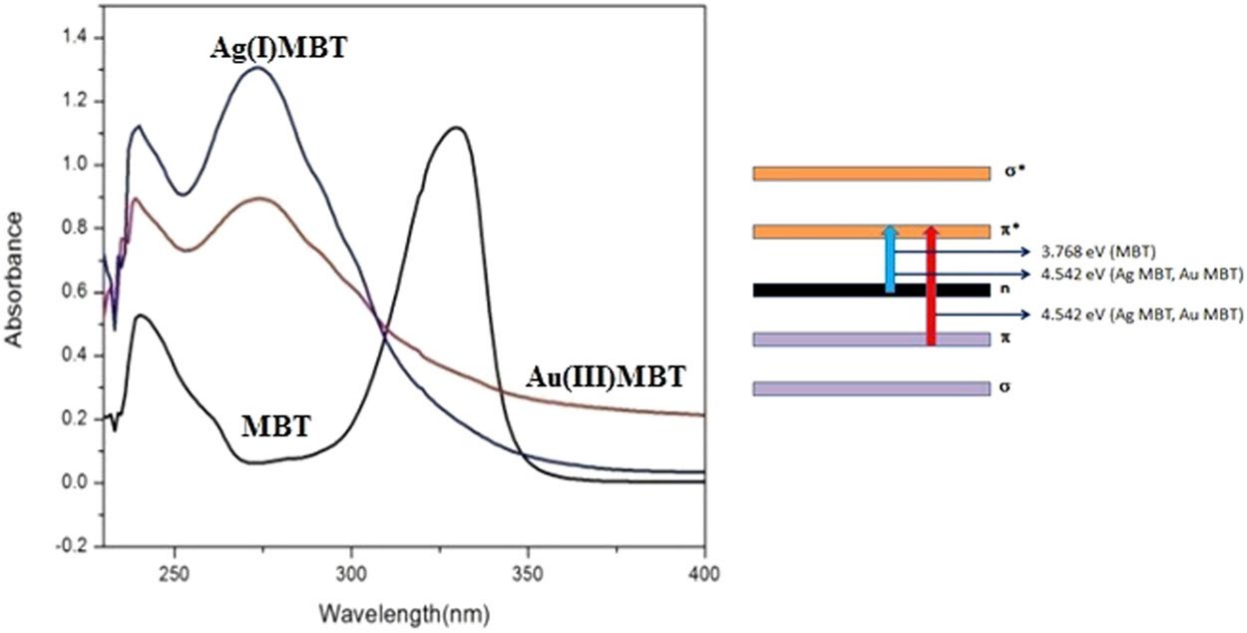
UV-Visible Spectroscopy of MBT, Au(III)MBT and Ag(I)MBT with the corresponding energy bad diagram.

The absorption spectra of MBT, Ag(I)MBT and Au(III)MBT was recorded in chloroform (Fig. 8). MBT showed an absorption spectrum at 239 and 329 nm. The peak at 239 nm (5.18 eV) correspond to π-π* transition of the aromatic moiety of the ligand which is not showing any shift in wavelength for both Ag(I)MBT and Au(III)MBT complexes whereas the peak at 329 nm (3.76 eV) attributed to n-π* transition of the heterocyclic ring system of the ligand, exhibits blue shift to 273 nm (4.54 eV) in both Ag(I)MBT and Au(III)MBT. This blue shift could be caused by the co-ordination of N and S donor atoms to Ag(I) and Au(III) metal ions. The intensity of the 239 and 273 nm peaks for Au(III)MBT is much lower than Ag(I)MBT. This indicates the existence of 1:1 ratio of metal cation to ligand in Au(III)MBT. The intensity ratio for 239 nm peak is *I*[Ag(I)MBT]/*I* [Au(III)MBT] = 1.1/0.9 = 1.22 whereas for 273 nm peak is *I*[Ag(I)MBT]/*I*[Au(III)MBT] = 1.31/0.88 = 1.49. As the intensity ratio is higher than unity in the case of Ag(I)MBT with respect to Au(III)MBT, more than one MBT moiety is coordinated with Ag(I) ion (cf. section 3.1). This is further supported by FTIR, ^1^HNMR and electrochemical analysis in the following sections.

#### Vibrational spectroscopy analysis

**Figure 9:**
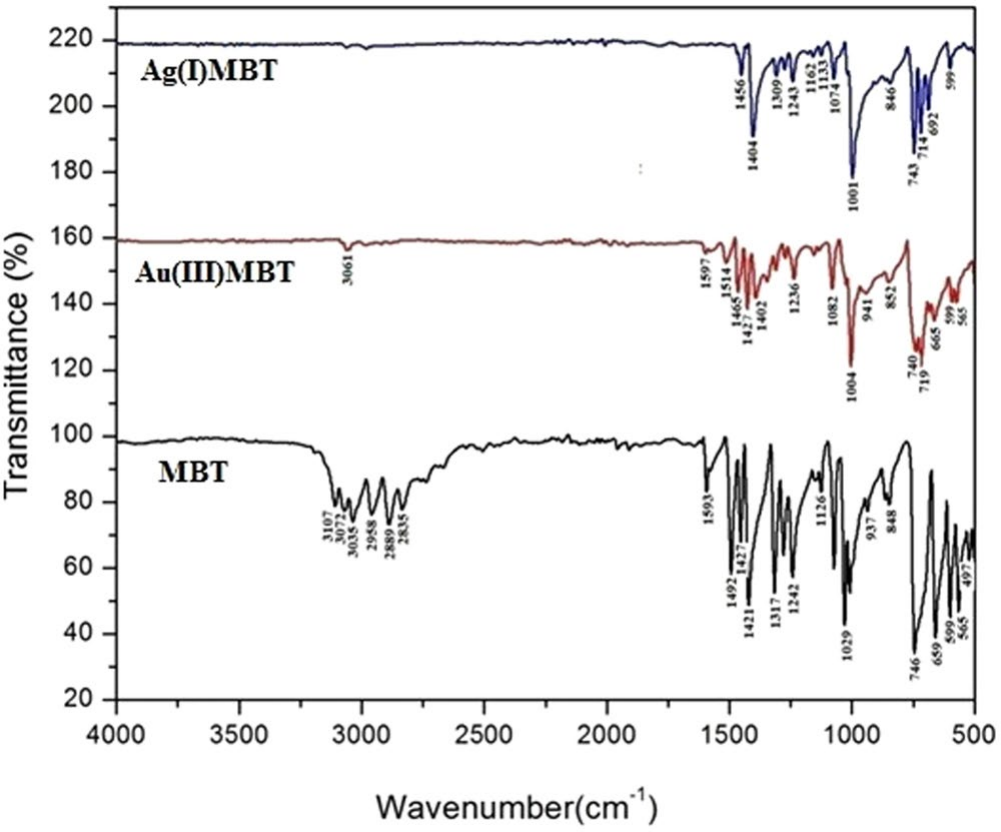
FTIR spectra of MBT, Ag(I)MBT andAu(III)MBT.

Figure 9 shows the FTIR spectrum of MBT, Ag(I)MBT and Au(III)MBT. The stretching vibration at 3072 cm^−1^ for MBT is attributed to aromatic C-H stretching which is red shifted to 3061 cm^−1^ in Au(III)MBT and completely absent in Ag(I)MBT. The selection criteria for IR active species state that “***vibrations involving dipole moments that are perpendicular to the surface only get excited***”^18^. As seen from section 3.1, due to the presence of aromatic C-H bond perpendicular to Au(III) ion, the complex Au(III)MBT obeys the selection rule and a C-H stretching peak is seen at 3061 cm^−1^, whereas in the case of Ag(I)MBT, the aromatic C-H stretching is parallel or in plane to the Ag(I) ion, therefore peak at 3061 cm^−1^ is not observed for Ag(I)MBT. Thus it can be concluded that MBT complexes with Ag(I) ion is in planar geometry and with Au(III) is in near perpendicular geometry. At lower wavenumber region, the in-plane C-C stretching at 1593 cm^−1^ for MBT is red shifted to 1514 cm^−1^ for Au(III)MBT and is very feeble for Ag(I)MBT. A vibrational frequency at 1427 cm^−1^ is observed for MBT which is attributed to ν(C-C) mode whereas this vibrational frequency is very strong in Au(III)MBT complex and absent in Ag(I)MBT complex. The ν(C-S) mode of vibration in MBT is observed at 599 cm^−1^ which remains very strong in both Au(III)MBT and Ag(I)MBT complex. The 692 cm^−1^ stretching vibration for Ag(I)MBT and 665 cm^−1^ stretching vibration for Au(III)MBT are due to C-S stretching of heterocyclic ring system, is not clearly seen for MBT due to absence of perpendicular orientation of the dipoles^3^. All the observed peaks in FTIR for MBT, Ag(I)MBT and Au(III)MBT is in agreement with the literature^17^. Thus FTIR analysis supports the structure of the complexes as shown in Fig. 7 of section 3.1.

### ^1^HNMR spectroscopy

**Figure 10:**
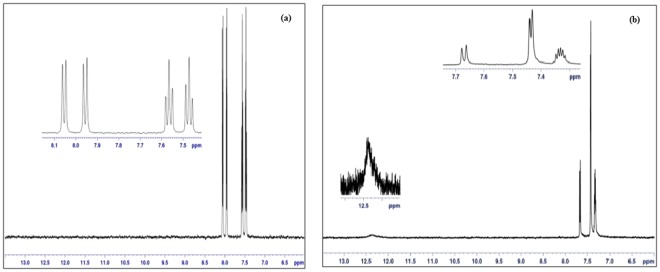
^1^H NMR spectra of (**a**) Ag(I)MBT (**b**) Au(III)MBT complex.

^1^HNMR chemical shifts were monitored to characterize Ag(I)MBT and Au(III)MBT (Fig. 10). Acetone-d_6_ solvent was used as the lock solvent. All the chemical shifts were referenced to the solvent peak at 2.8 ppm. The experiment was carried out at 300 K over a spectral width of 15.0 ppm. The ^1^H NMR chemical shifts for Ag(I)MBT and Au(III)MBT complexes are as follows:

1. Ag(I)MBT: ^1^H NMR(aetone-d_6_)δ 7.48 t, H(Ar); 7.57 t, H(Ar); 7.95 d, H(Ar); 8.05 d, H(Ar).
2. Au(III)MBT: ^1^H NMR(acetone-d_6_)δ 7.34 m, H(Ar); 7.44 d, H(Ar); 7.68 d, H(Ar); 12.39 s, H(SH).

**Figure 11:**
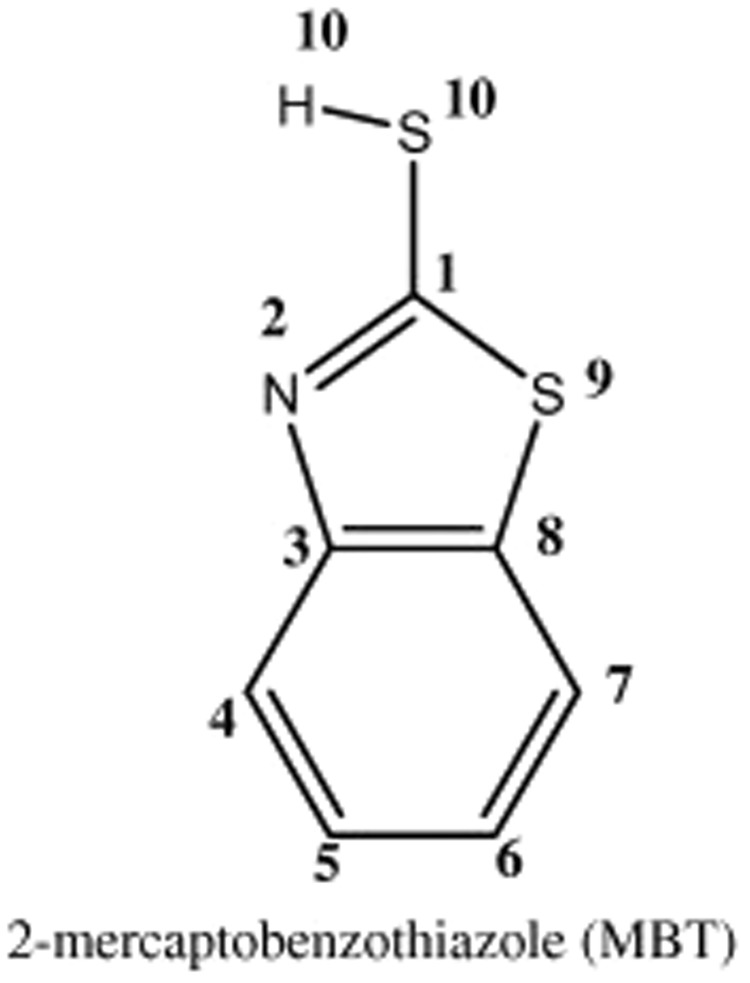
Labelled structure of Mercaptobenzothiazole.

The ^1^HNMR spectra of both the complexes exhibit 1H peaks related to four aromatic protons present in the benzene ring of the mercaptobenzothiazole (Fig. 11). A closer inspection of the ^1^HNMR data revealed that in case of Ag(I)MBT all the aromatic protons are shifted to high frequency (downfield) compared to that of the Au(III)MBT complex. There is a pair of deshieled doublet pattern observed in both the complexes corresponding to protons attached to C4 and C7. In case of Ag(I)MBT there are two triplets at 7.48 and 7.57 ppm while the triplets have merged together and observed as a single multiplet pattern at 7.34 ppm in case of Au(III)MBT complex. Further, the –SH proton peak is missing in case of Ag(I)MBT which is clearly observed in case of Au(III)MBT as a broad singlet at 12.39 ppm. This observation can be attributed to the fact that in case of Au(III)MBT complex sulphur has not coordinated to the Au(III) ion and remains as –SH whereas in case of Ag(I)MBT, the –SH proton is involved in the complex formation via coordinating with the Ag(I) ion. Such coordination has also brought an overall deshielding effect for all the aromatic protons of Ag(I)MBT. On the basis of NMR analysis, we tried to support that complexation of silver with MBT has happened through –SH group, while in case of gold the –SH group remains intact. Further, the aromatic protons of MBT experienced a greater downfield shift in case of silver complex compared to that of the gold complex. We must clarify that we have not tried to determine the complex structure using NMR data, rather we have used NMR to identify the event of complexation and the different mode of complexation in case of Au(III)MBT and in case of Ag(I)MBT. Thus the structure shown in Fig. 7 of section 3.1 is supported by UV-Visible, FTIR and ^1^HNMR studies.

### Inductively coupled plasma mass spectroscopy (ICP-MS) analysis

ICP-MS was used to monitor the concentration of metal ion in Ag(I)MBT and Au(III)MBT complexes. The ICP-MS analysis confirmed the concentration^19^ of Au and Ag in the electrolyte as 2.5 g/dm^3^. The data obtained for the complexes showed the presence of 15% of Ag in 0.0410 grams of Ag(I)MBT sample and 10% of Au in 0.0129 gms of Au(III)MBT sample, thus confirming that the synthesized complexes are highly pure with only Ag(I) coordinating to the ligand MBT in Ag(I)MBT complex and Au(III) coordinating to the ligand MBT in Au(III)MBT complex.

### Electrochemical studies

**Figure 12:**
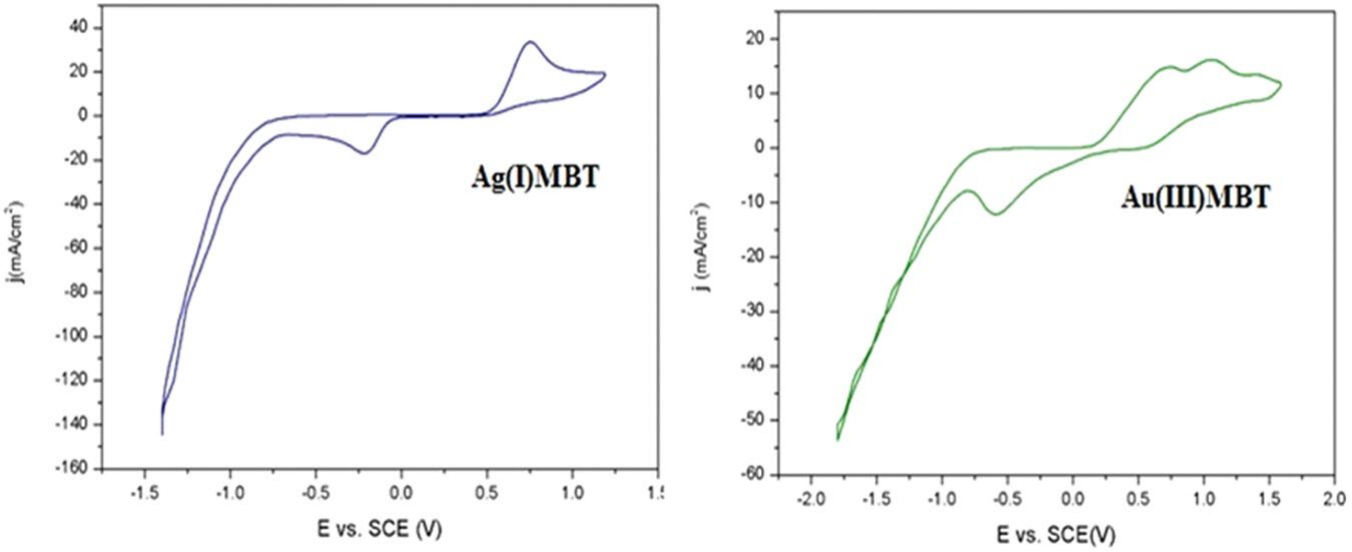
Cyclic voltammogram of Ag(I)MBT and Au(III)MBT at 50 mV/s.

Electrochemical studies were carried out using cyclic voltammetric technique employing Zahner Zennium electrochemical workstation. In a standard three-electrode electrochemical cell, the working electrode was a gold electrode (surface 0.02 cm^2^), whose potential was controlled against the saturated calomel reference electrode (SCE). Platinum coil served as a counter electrode. Figure 12 indicates the cyclic voltammogram of Ag(I)MBT and Au(III)MBT at pH 5. For Ag(I)MBT, peaks corresponding to anodic and cathodic reactions are noticed. Cathodic peak at 0.746 V indicate reduction of Ag(I) ion into Ag and anodic peak at −0.3 V indicate the formation of Ag-S bond with MBT rather than reversibly oxidizing to Ag(I) ions. This is as anticipated in the cellular pH. It is well demonstrated in the literature^20^ that upon treating the cells with silver complexes, pH of the cell becomes acidic (near 5) thereby reducing Ag(I) to Ag and subsequently inducing cell apoptosis. The reduction of Ag(I) to Ag at pH 5 is supported by cyclic voltammetric studies. In the case of Au(III)MBT, as the complex is expected to exist as ion pair (cf. sections 3.1, 3.2 and 3.3), Au(III) ion exists as AuCl_4_^−^ and hence three cathodic peaks are noticed in Au(III)MBT voltammogram at 0.45, 1.0 and 1.5 V. The first peak corresponds to the redox reaction Au^3+^ + 2e → Au^+^, the second peak accounts for AuCl_4_^−^ + 3e → Au and the third peak indicates the formation of Au^3+^ + 3e → Au. This demonstrates the presence of AuCl_4_^−^ anion and free Au^3+^ ions in the solution. Anodic peaks were noticed at 0.6  and – 0.6 V. This is due to the oxidation of Au^+^ ions released in the solution back to Au^3+^ ions, as Au^3+^ is the most stable oxidation state for Au. Thus in addition to UV-Visible, FTIR and ^1^HNMR studies, electrochemical analysis of Ag(I)MBT also support the formation of S coordinated Ag(I) ions and ion pair complexes in Au(III).

### Cell Culture and TUNEL Analysis

**Figure 13:**
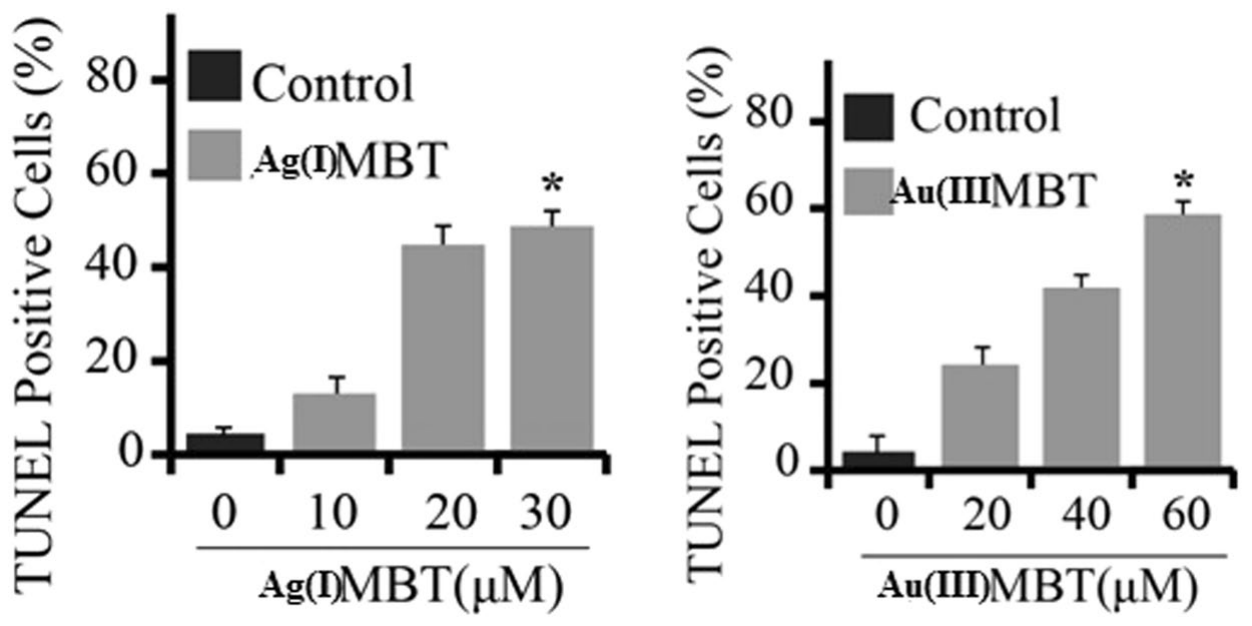
TUNEL analysis of A549 cells using Ag(I)MBT and Au(III)MBT.

**Figure 14:**
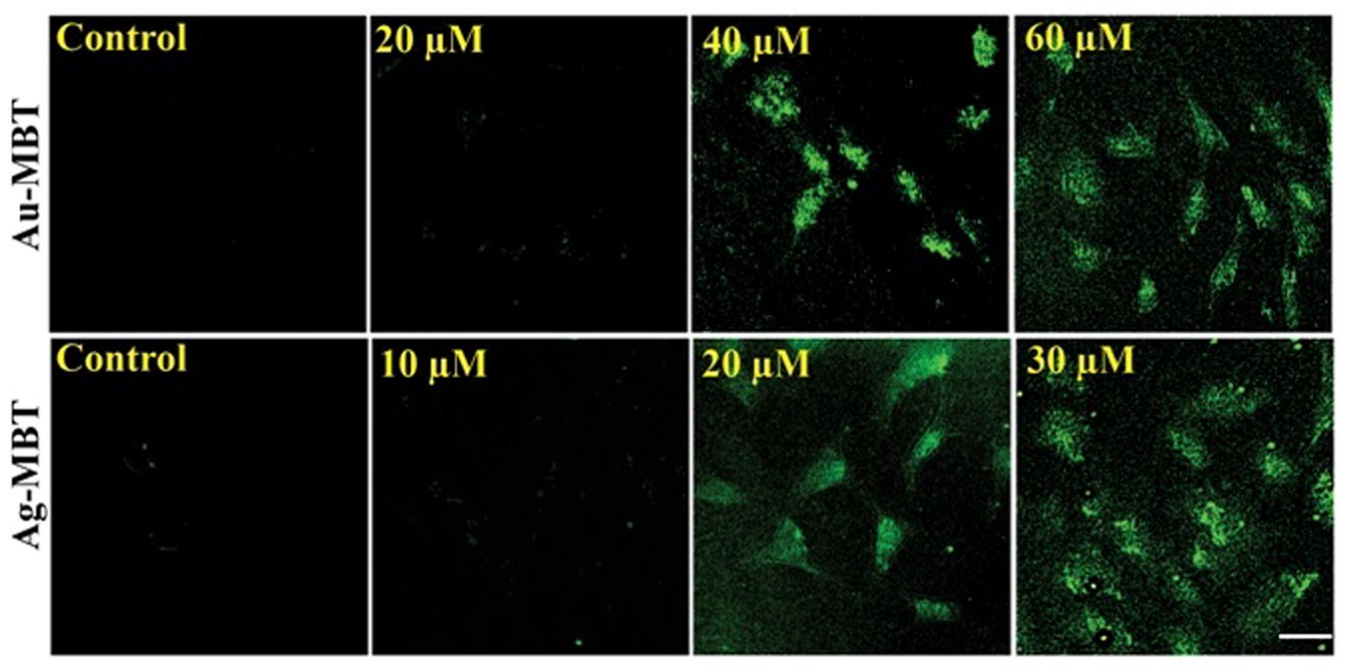
Fluorescent microscope image TUNEL detection of A549 cells apoptosis after Au(III)MBT and Ag(I)MBT DCA treatment in concentration dependent manner.

During apoptosis, DNA cleaves to generate myriad fragments with double stranded and single stranded nick in the nucleus^21,22^. The fragmentation of DNA during apoptosis are labeled *in-situ* by attaching fluorescent-tagged nucleotides into partially degraded DNA using terminal deoxynucleotidyl transferase (TdT) and DNA polymerase^23,24^. The tail labeling reaction was done using TdT^25–29^. This assay is commonly known as TUNEL ‘TDT-mediated dUTP-biotin nick-end labelling’^30^. Different variations of this assay have been developed^29^. Amongst all variants, the assay based on incorporation of BrdUTP seems to be more promising in terms of simplicity and sensitivity^28^. In this assay FITC-conjugated anti-BrdU attaches to poly BrdU found at the site of DSBs^31^. DNA denaturation is not required for the attachment of antibody to poly BrdU in DSB but it is required to detect the monomer incorporation during the process of DNA replication^31,32^. To perform this assay prefixation of cells using formaldehyde was done to retain the oligomeric fragments inside the cell. Labelling of nicks with FITC-tagged anti-BrdU antibody can be combined with red fluroscence staining of DNA. These cell subpopulations were analyzed under multiparameter cytometer to differentiate the cell which undergo apoptosis process from non-apoptotic subpopulations^25,26^. A549 cells were platted into six well tissue culture plates for various treatment experiments and on subsequent day at 70–80% confluence. From the TUNEL^14^ assay analysis of Ag(I)MBT and Au(III)MBT treated cells, we observed a new crucial insight into the mechanism. The probability of induced apoptosis in A549 cells upon treatment with both Ag(I)MBT and Au(III)MBT had been noticed (cf section 3.8). A549 cells were seeded into six wells tissue culture plates and twenty wells were treated with or without (DMSO) as control experiments. Then the cells were treated with Ag(I)MBT (Fig. 13a) and Au(III)MBT (Fig. 13b) complexes at different concentrations ranging from 0 to 40 μM for Ag(I)MBT and 0 to 80 μM for Au(III)MBT. After 24 hrs of the treatment, post-treated cells were processed for detection of apoptosis in the terminal via deoxynucleotidyl transferased UTP nick-end labeling (TUNEL) analysis^14^. Figure 13 depicts the bar chart of the TUNEL analysis with mean ± SD of three independent experiments which were performed in triplicates and *p < 0.05 as compared to control cells. The TUNEL assay studies in conjunction with the Fluorescence microscope images of A549cell line treated with Ag(I)MBT and Au(III)MBT complexes indicated induced apoptosis in the cells (Fig. 14). TUNEL assay showed 60% cell viability for Ag(I)MBT at concentration of 30 μM whereas 80% for Au(III)MBT at 60 μM. Thus Ag(I)MBT can induce cell apoptosis in A549 cell line at a higher rate and lower concentration than Au(III)MBT. The cell viability was achieved for Ag(I)MBT at half the concentration of Au(III)MBT. Therefore, these complexes studied here can be a viable option as anti – proliferative agent.

To understand cell viability effects of Ag(I)MBT and Au(III)MBT complexes, we have performed MTT assay. The viable cells can change MTT to colored formazan crystals which are imaged by bright field image microscopy. Cells were platted into 96 well plates and next day they were treated with appropriate doses of Ag(I)MBT and Au(III)MBT complexes as shown in Supporting Information. MTT solution was used by diluting 5 mg of MTT reagent in 1 ml of PBS and incubated to cell samples for 4 hours at 37 °C in dark. One hour incubation at 37 °C in dark was given after addition of acidic isopropanol and proper mixing to dissolve formazan crystals. Absorbance were monitored at 570 nm wavelength with the help of microplate reader. As there is no additional data to support the proposed mechanism, we have performed the detailed bright field image microscopy and counted the cell numbers manually. The data clearly represent the overall good health and fitness of the cells. We have observed the difference between the control (non-treated), Ag(I)MBT and Au(III)MBT complexes treated samples in terms of cellular proliferation (cf. Supporting Information).

### Plausible mechanistic pathway for Ag(I)MBT and Au(III)MBT based apoptosis of cell

**Figure 15:**
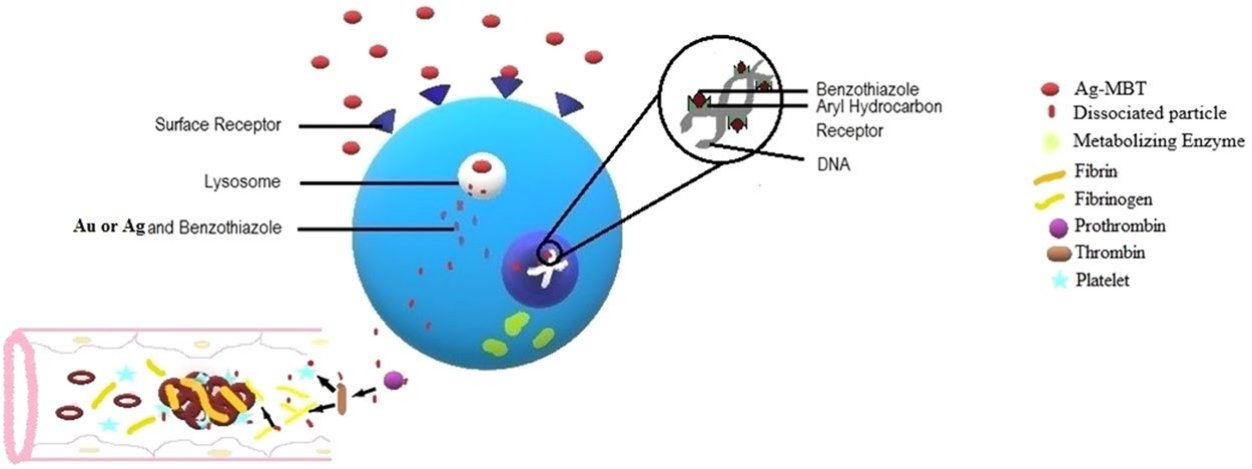
Plausible mechanistic pathway for apoptosis of the cell treated with Ag(I)MBT or Au(III)MBT.

Ag(I)MBT or Au(III)MBT get attached to cell surface receptor and get engulfed as a vesicle carrying the complex, which forms an early endosome. Once the early endosome gets attached to lysosome, its pH reduces^20^ to form late endosome (ca. pH = 5.5). As a consequence of reduced pH, Ag or Au gets released from the complex. MBT metabolizes to benzothiazole and H_3_S^+^ which makes the lysosomal environment even more acidic. This strong acidic environment facilitates the release of Ag or Au and benzothiazole out of late endosome. The escaped Ag or Au and benzothiazole get into the nucleus and damages the DNA which leads to the induction of apoptosis^20,33,34^ (cf. Fig. 15). Analogous to the proposed mechanism, Ag(I)MBT is reduced to Ag in acidic pH leading to degradation of Ag(I)MBT complex when electrochemically perturbed. This unique observation in the cyclic voltametric analysis of Ag(I)MBT and Au(III)MBT implicitly support the formation of free Ag and Au in cells at acidic pH of 5.5.

## Perspectives and Summary

Ag(I)MBT and Au(III)MBT synthesized via wet chemical method is characterized for optical, structural, electrochemical properties. The structural and optical studies confirmed the formation of linear complex for Ag(I)MBT and near perpendicular complex for Au(III)MBT. ^1^HNMR studies also supported the linear and perpendicular structure of Ag(I)MBT and Au(III)MBT complexes. The electrochemical analysis at acidic pH of 5 showed release of Ag^+^ ions at cellular pH. This released Ag^+^ is reduced to metallic silver in the cathodic scan, whereas Au^3+^ reduces to Au^+^. This unique observation from electrochemical analysis supported the plausible mechanism of apoptosis in cells by Ag(I)MBT complex. The TUNEL and MTT assay on A549 cells and control cells revealed induced apoptosis and cellular anti-proliferation. Thus the complexes studied in the present investigation can be a viable option as anti-proliferative agent.

## Electronic supplementary material

Supplementary Information
