# Supplementary material for: Ag(I) and Au(III) Mercaptobenzothiazole complexes induced apoptotic cell death
Source: Sci Rep. 2019 Jan 24;9:621. doi: 10.1038/s41598-018-36801-6 (PMC6345975; doi:10.1038/s41598-018-36801-6)
Supplement: Supplementary file 1 — Supplementary Information [file 41598_2018_36801_MOESM1_ESM.docx]

**Ag(I) and Au(III) Mercaptobenzothiazole complexes induced apoptotic cell death**

Jositta Sherine^a^, Arun Upadhyay^b^, Amit Mishra^b^, Deepak Kumar^c^, Samanwita Pal^c^, S. Harinipriya^d*^

^a^*Department of Physics and Nanotechnology, SRM Institute of Science and Technology, Kattankulathur, India, 603203*

*^b^Cellular and Molecular Neurobiology Unit, Department of Biology, Indian Institute of Technology Jodhpur, Rajasthan, India, 342011*

*^c^Department of Chemistry, Indian Institute of Technology, Jodhpur, Rajasthan, India, 342011*

*^d*^Corresponding Author,Electrochemical Systems Lab, SRM Research Institute, SRM Institute of Science and Technology, Kattankulathur, India 603203*

**
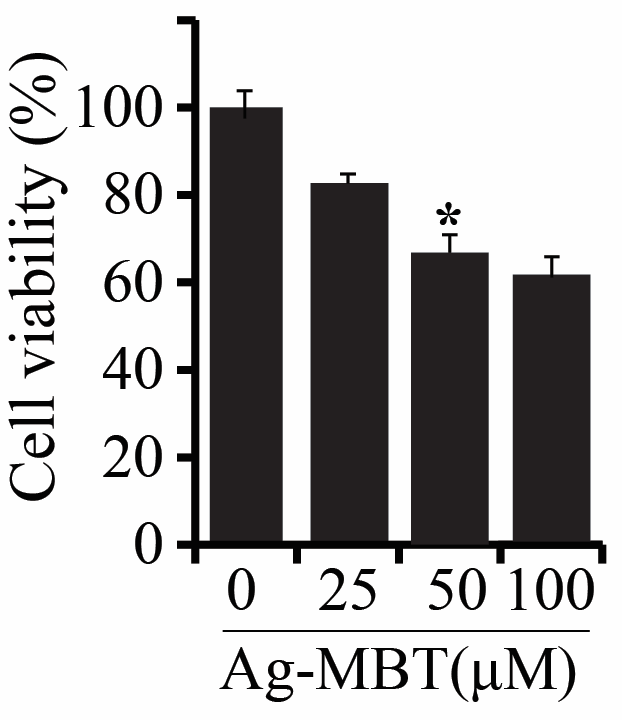

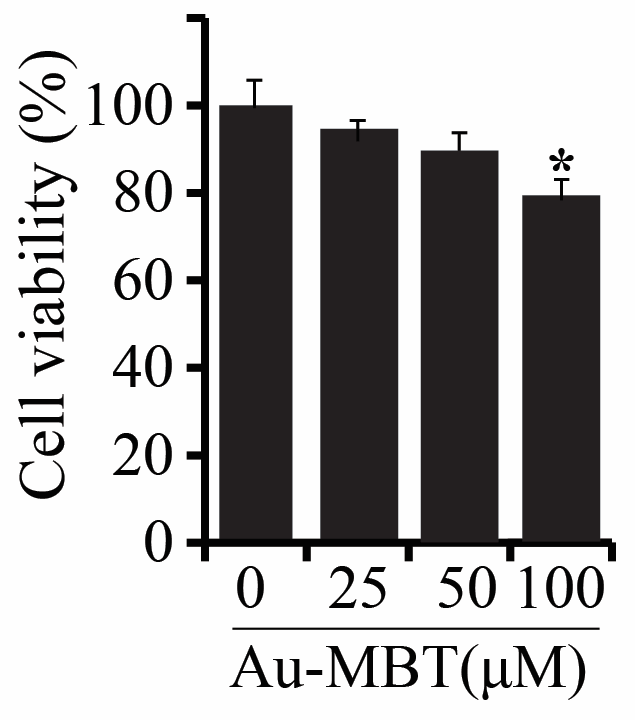
**

**Materials and Methods**

***Cell Culture, MTT Assay and Bright Field Image Analysis****:*

Cell culture and other biochemicals were procured from Sigma. The A549 cells were grown in DMEM containing 10% horse serum, 5% fetal calf serum, and penicillin/streptomycin in 5% CO_2_. For MTT, 3-(4,5-dimethylthiazol-2-yl)-2,5-diphenyltetrazolium bromide; assay cells were passaged into 6-well tissue culture dishes. The following day, cells were rinsed and the medium was changed and after six hours, Ag-MBT and Au-MBT were added, and 24 hours later cell viability was assessed by MTT assay. Cells were also exposed without (control) or with Ag-MBT and Au-MBT at different concentration and after treatment of 48 hours cells were analysed under bright field image microscope.

**Figure Legend**

**Figure (A-B).** As shown in figure cells were plated into six well culture plates and after 24 hours treated in the absence (DMSO) or presence of Ag-MBT (A) and Au-MBT (B) compounds at concentration dependent manner. Post-treated cells were used MTT assay for cell viability analysis. Values are means ±S.D. of three independent experiments, each performedin triplicate. *, p < 0.05compared with the untreated and treated (Ag-MBT and Au-MBT) groups.

**
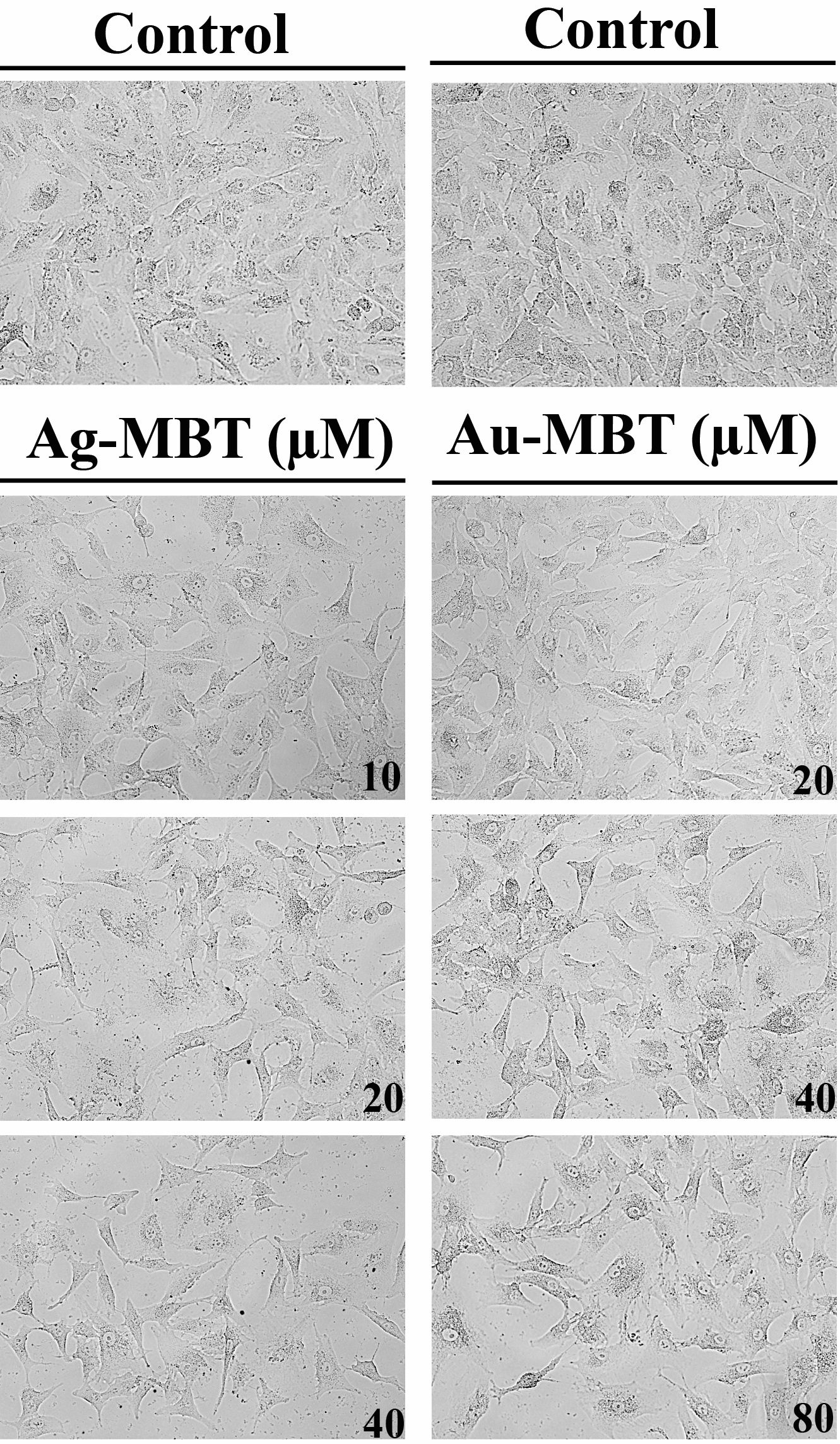
**

**Figure Legend**

**Figure:** Cells were treated with (Ag-MBT and Au-MBT)groups at various concentrations and few similar sets of cells were not exposed with above compounds and used them as control. Post-treated cells were subjected to bright field microscopy observation as shown in micrographs analysis

**Results:** The cell viability was measured by MTT assay analysis of Ag-MBT and Au-MBT-treated cells. The results in figure represent that Ag-MBT and Au-MBT treatment reduces cell viability as compared to control cells. Bright field image analysis reveals that treatment of Ag-MBT and Au-MBT is non-toxic in nature and cells are overall healthy at different concentrations; overall cellular proliferation rate is slow in Ag-MBT and Au-MBT treated cells as compare to normal (untreated) cells.
